# Male Nursing Officers’ Perceptions and Experiences of Workplace Gender Inequality in Odisha, India: A Qualitative Study

**DOI:** 10.7759/cureus.90994

**Published:** 2025-08-25

**Authors:** Sohini Karmakar, Trupti Swain, Anasuya Pattanayak, Soumya Sonalika, Krushna Chandra Sahoo

**Affiliations:** 1 Community Health Nursing, Kalinga Institute of Nursing Sciences, Bhubaneswar, IND; 2 Public Health, ICMR-Regional Medical Research Centre, Bhubaneswar, IND

**Keywords:** experience, gender inequality, male nursing officers, perception, workplace

## Abstract

Aim and objective

This study sought to explore male nursing officers’ perceptions and experiences of workplace gender inequality in Odisha.

Design

This study employed a qualitative study design using the descriptive phenomenological approach.

Methods

Data were generated by individual face-to-face interviews among 14 male nursing officers who had a clinical experience of two or more years and worked in tertiary care hospitals in Odisha. The COREQ criteria and checklist were followed during the study, and data were analyzed using the MAXQDA software.

Results

Based on the findings, three themes were extracted that described male nursing officers’ perceptions and experiences of workplace gender inequality. In the first theme, “Current opportunities in clinical settings,” male nursing officers’ diverse range of skills and specializations were stated. The second theme, “Challenges and differences due to gender,” illustrated the workplace prejudice and discrimination that a male nursing officer had to cope with. The third theme, “Initiatives and suggestions to enhance the numbers of male nursing officers,” highlighted the Odisha government’s effort to end discrimination and uplift the stature of male nursing officers in the society.

Conclusion

Gender inequality experienced by male nursing officers in workplace arise from patients, co-workers, and higher authorities. This can be resolved by avoiding classifying certain roles according to the gender, providing equal posting in all areas regardless of gender, and raising societal awareness about male nursing officers and their obligations.

## Introduction

To fulfill the responsibility of "leaving no one behind" and the global endeavor to achieve sustainable development goals, nursing officers are indispensable [1]. A case study by experts at the International Council of Nurses highlighted that women constitute the majority of nursing officers, with only a small proportion being men [2]. In India, male nursing officers have a long-standing history - the world’s first nursing school, established in 250 BC, was open exclusively to men. A recent study revealed that Indian male nursing officers were particularly active in the early 20th century, especially in the decades preceding independence. Globally, male nursing officers make up only a small fraction of the nursing workforce, with women comprising approximately 90% [3]. This gender imbalance is concerning, as it is vital for any profession to reflect the demographics of the general population [4].

Male nursing officers: the divergent term

Historically, men were involved in caring for the sick, injured, and dying as part of religious organizations following the introduction of Christianity, which promoted a compassionate ethos. During this time, both men and women provided nursing care, though male nursing officers primarily cared for male patients, alcoholics, the mentally ill, and violent individuals [2]. Research indicates that male nursing officers are often stereotyped and used for practical demonstrations, which is perceived as more acceptable than using female counterparts. This contributes to feelings of shame and declining interest in the profession among male nursing officers [2]. A lack of visible male role models in the media and prevailing negative societal perceptions, such as the belief that nursing is a "female" profession, have created significant barriers to male enrollment [3-5]. The shortage of male nursing officers also limits gender diversity in healthcare teams, depriving patients of a holistic and inclusive care environment. A 2018 study reported that only 9.6% of nursing officers are male, highlighting a stark gender disparity [5-7]. Many have voiced frustration at the assumption that nursing is a fallback career after unsuccessful attempts to become a doctor, further lowering their motivation and professional identity [8]

Men in nursing: challenges and advantages

Choosing a profession that is not traditionally associated with men exposes male nursing officers to social stigma, which hinders their ability to provide optimal care [9]. This begins in the classroom, where male nursing students are typically a minority. Many male students and professionals report experiencing rejection, discrimination, and false accusations by patients and families. They also face inadequate support from female colleagues, nurse managers, and educators, along with a lack of male mentors [10]. These conditions contribute to unmet learning needs and internal doubts about their professional identity and career trajectory [11]. The Central Administrative Tribunal (CAT) has issued notice to the Center and the All India Institute of Medical Sciences (AIIMS) on a plea to quash the decision of the premier institute to reserve 80% of seats for women for filling vacancies of nursing officer [12]. Such challenges influence male nursing officers’ decisions to remain in the profession and affect their professional relationships, self-perception, and opportunities for advancement in clinical and leadership roles [13]. Despite these barriers, male nursing officers often serve as role models for other men, inspiring more male participation in the profession. This representation has also supported some in rising to higher-paying and more prestigious positions within the nursing hierarchy [14-15].

Current status of nurses worldwide

Globally, there are approximately 27.9 million nursing officers, of whom 19.3 million are certified professionals. Between 2013 and 2018, the workforce grew by 4.7 million, confirming that nursing is the largest occupational group in healthcare, comprising nearly 59% of all health professionals. However, only around 10% of the global nursing workforce comprise men [15-16]. In India, the nursing workforce is facing a severe shortfall of around 2 million, which represents a 43% deficit compared to the World Health Organization’s recommended threshold [17-18].

This study aims to explore the perceptions and experiences of male nursing officers regarding gender inequality in clinical practice in Odisha, India, an area where no prior studies have been conducted.

## Materials and methods

Design

Data were obtained through a phenomenological descriptive design with a qualitative methodology. The phenomenological approach offered the most effective way to articulate and comprehend the male nursing officers’ perceptions and experiences in clinical settings from their viewpoints.

Study setting

One public tertiary care hospital and one private tertiary care hospital in the Khordha district of Bhubaneswar, Odisha, were specifically chosen for this study. These tertiary care hospitals were chosen for their proximity, convenience, and feasibility.

Participants

Male nursing officers working in the selected public and private tertiary care hospitals were selected as the study population. A total of 14 male nursing officers (4 from the government hospital and 10 from the private hospital) working in the chosen tertiary care hospitals were selected as the study population using a purposive sampling technique. To guarantee an equitable distribution of participants between government and non-government institutions, 29 male nursing officials were contacted; however, 15 participants declined to participate due to their busy schedules. Male nursing officers with a clinical experience of two or more years were selected for this study. Prior to the interview, no personal connection made with the participants, and the first author contacted each participant either personally or over the phone.

Data collection procedures

Data were gathered through in-depth interview using the semi-structured open-ended interview guide to explore the perceptions and experiences of male nursing officers working in the clinical settings (Table 1). The first author conducted each interview in the languages that the participants were comfortable with (“Odia” and “Hindi” languages in this case), and each interview lasted for an average of 40 minutes. The interviews were not conducted again. After 12 interviews, the data became saturated, but the first author conducted two more interviews. The first author is female, with an educational background in nursing sciences, and certified in qualitative research techniques. The co-authors have different backgrounds: biological and environmental sciences, as well as experience in social services and community health nursing.

**Table 1:** In-depth interview guide

Interview guide
How would you describe the position of male nursing officers in the society? Probes: trolled for choosing a female profession, served as “living example” of success, role models, nursing is an option for men who have not been able to pursue studies in medicine or dentistry.
Nursing is considered a female profession - what are your thoughts regarding this statement? Probes: motherly attitude, more empathetic than males.
What are the challenges faced by the male nursing officers related to promotion and career development? Probes: fewer opportunities, denied chances for seminars/workshops, further training, assigned tiresome and difficult tasks.
Share your work experiences with the female patients. Probes: difficult and challenging during the intimate care, trust issues of the husband/family member.

Data management and analysis

The verbatim transcriptions of the recorded interviews were made. The transcripts were converted into English and analyzed utilizing the content analysis method. The transcripts were accessible only to the first author. The transcripts were not sent back to the participants for modifications or comments. In order to verify the accuracy of the transcripts, verbatim transcription of the interviews was performed after the acquisition of raw data. The transcript’s authenticity was thoroughly reviewed. The meaning units were abridged and encoded by three authors. Clusters of related codes were condensed into subcategories and categories. The themes were generated by utilizing the developed codes. To ensure that the themes were accurate and usable representation of the data, the themes were reviewed by all the authors. The text segments were coded and categorized using the MAXQDA software.

Ethical approval

The Institutional Ethical Committee granted approval for the study. Permission from the public and private tertiary care hospitals was obtained prior to the study’s execution. The first author explained the study’s objective to the participants, and their written consent was obtained. With the consent of all participants in the interviews, every interview was digitally recorded, and field notes were taken. The recordings were accessible only to the first author via a password-protected file. Every interview was conducted with complete secrecy at a site chosen by the interviewee, and participants’ identities were kept confidential.

## Results

A total of 14 male nursing officers between 24 and 35 years of age were interviewed. Their range of nursing experience was 2 to 10 years. The educational level of the majority of the participants was GNM (general nursing and midwifery), and among them, four participants had a B.Sc. degree (bachelor in nursing) and three participants had a PBB.Sc. degree (post basic bachelor of science in nursing). The participants had work experience in general OT (operation theater), O&G OT (obstetrics and gynecology operation theater), ICU (intensive care unit), MICU (medical intensive care unit), emergency, Cath lab (cardiac catheterization lab), psychiatric ward, gastroenterology ward, and surgery ward. Three major themes emerged from the analysis: “current opportunities in clinical settings” (Table 2), “challenges and differences due to gender” (Table 3), and “initiatives and suggestions to enhance the numbers of male nursing officers.”

**Table 2:** Coding tree of theme 1 (categories and codes)

Theme 1: Current opportunities in clinical settings					
Categories	Diversity	Specializations	Career Advancement	Educational Initiatives	Global Trends
Examples of code	Work as a member of the rapid response team	Preferred into some certain area, such as OT, ICU, emergency, Cath lab, psychiatric ward, and critical care unit	Promoted quickly in some private hospitals	Acts as a “role model” and inspire the newcomers	Demand of male nursing officers more in current scenario

**Table 3:** Coding tree of theme 2 (categories and codes)

Theme 2: Challenges and differences due to gender					
Categories	Gender Stereotypes	Social Stigma	Workplace Bias	Patient Preferences	Less Scope
Examples of code	Considered as a female-dominated profession	Questions regarding job description	Other healthcare professionals behave differently due to gender bias	Awkwardness due to gender and maintenance of privacy	Lack of skills due to no posting in the O&G department or NICU

Theme 1: current opportunities in clinical settings

A wide range of patient care concerns have been handled by nurses. Few professions have such a diverse range of skills or obligations. In addition to the extensive variety of routine tasks, there are numerous specializations and work arrangements available to the male nursing officers.

Category 1: Diversity

Male nursing officers offer high-quality, unique nursing care to the patients. Nursing officers who demonstrate strong technical proficiency and a keen interest in learning new technology are better suited for roles that require these skills. Many of the participants stated that they are essential to strengthening the healthcare system by being part of the rapid response team (RRT) and being available at any time (24x7) to respond to emergencies. By being accessible during the night for emergencies, they are also relieving pressure and supporting their female counterparts, as it is difficult for females to reach to the hospital at midnight.

“When I was caring for an elderly patient, she addressed and blessed me by saying “beta,” at that time I felt very delighted, and this motivated me to provide better nursing care to the patients.” (Participant 2)

“Male nursing officers have a positive attitude, an interest in technology, and more knowledge about its applications. When a new instrument arrives in the ward, it is usually first assigned to the male nursing officers.” (Participant 10)

“In an emergency situation, male nursing officers can go to hospitals at night, but this is problematic for female officers.” (Participant 8)

Category 2: Specializations

Opportunities in areas such as the critical care unit, emergency, OT, ICU, and psychiatric ward are often based on the required expertise in specific procedures, as well as the ability to handle physically demanding tasks and contribute diverse perspectives, which can be advantageous for patient care.

“I’m an expert in suturing and assisting, with experience in CTVS OT.” (Participant 3)

“I’m confident that in the psychiatric ward, I manage the patients more skillfully than the female nursing officers.” (Participant 4)

Category 3: Career Advancement

To overcome the challenges, some healthcare institutions recognize the value of male nursing officers and actively work to promote gender equality in the profession, offering career advancement opportunities. In certain private hospitals, nursing officers are promoted based on specific skills, competencies, and unique professional attributes that align with organizational needs.

“I frequently received praise for my work from my in-charges, supervisors, and hospital authorities, and they are also planning in-service program to give us up-to-date knowledge.” (Participant 9)

“The supervisors preferred male nursing officers over female nursing officers because they completed their work quickly and on schedule.” (Participant 6)

Category 4: Educational Initiatives

Male nursing officers also serve as role models and inspire more men to pursue nursing as a profession, leading to increased diversity in nursing schools and the healthcare sector. Furthermore, they inspire junior male nursing officers and male nursing students. Male nursing officers often encourage newcomers and adopt a positive approach when imparting knowledge and skills to juniors.

“When I was a student, a brother encouraged me, and he was my role model.” (Participant 2)

“Male nursing officers are helping the newcomers to learn skills considerably more quickly and easily than female nursing officers.” (Participant 14)

Category 5: Global Trends

The global nursing landscape is evolving, and more men are entering the profession worldwide. In the current situation, both hospital administration and patients necessitate the male nursing officers in the provision of care. Most of the students, regardless of gender, choose this profession in order to have a better future.

“In the current scenario, everyone is entering this profession, regardless of gender.” (Participant 1)

“By joining this profession, some of the male nursing officers also influence the society by acting as a changing agent.” (Participant 11)

Theme 2: challenges and differences due to gender

The clinical environment produces a variety of difficulties and challenges for male nursing officers. In a largely female-dominated profession, male nursing officers face stereotyping, discrimination, and difficulties in finding mentors and role models who can guide them in navigating their career.

Category 1: Gender Stereotypes

In India, nursing was traditionally seen as a profession dominated by women. Male nurse officer’s service and contributions are not appreciated in society as a result of a lack of awareness about their tasks and responsibilities. Due to the gender of male nursing officers, society and the general public make fun of them. Participants mentioned that the society perceives nursing as a female profession and they are frequently questioned about their career choices.

“The public is unaware about the male nursing officers’ roles and responsibilities because we are not well-known in the society.” (Participant 3)

“In the society, nursing is considered as a female profession; I think this ‘taboo’ should be removed.” (Participant 10)

“When I joined this profession, I experienced questions like, ‘being a male how you got admission in this profession?’; ‘Do you think you can do this work?’; ‘Why did you studied nursing?’” (Participant 7)

Category 2: Social Stigma

Male nursing officers frequently encounter questions about their job descriptions. In clinical settings, the male nursing officers are mostly referred to as "doctors," "pharmacists," or "technicians." Most of the people refuted the existence of the post of male nursing officer, and in remote areas, the society referred them as "compounders” due to the lack of knowledge and awareness. They also experienced trouble finding a "partner" for marriage and occasionally encounter rejection and hostility from the girl’s family due to their profession.

“People think that nursing is all about giving tablets and injections.” (Participant 4)

“There is a ‘stigma’ in the society, people think we have no standards and we are only giving tablets and injections. People verbally torment us and often physically assault us.” (Participant 11)

“Most of the people considered me as an other healthcare professional rather than nursing professional. People often asked me, ‘Are you a doctor or pharmacist?’; ‘What is your post?’ Sometimes they claimed that there is no such post as male nursing officer and that there are no males in the nursing profession.” (Participant 2)

“I observed that my in-charge experienced problem regarding marriage. He belongs to Kerala and there it is a respectful profession for both boys and girls. That time, he was in love with a girl, and when he proposed her for marriage, the girl’s parents rejected him by saying, we can’t accept that our son-in-law is a male nursing officer.” (Participant 10)

Category 3: Workplace Bias

Since there are more female nursing officers in the clinical settings, they are given priority for promotions. Male nursing officers have less opportunities for higher positions, which restricts their prospects for advancement. Due to their muscular physique, they are often referred as “power lifters” or “helping hands” in healthcare settings.

“Because there are more female officers than male nursing officers, women receive promotions faster.” (Participant 5)

“The female nursing officers got promotion easily, because they maintain very good communication with the nursing in-charge; but we don’t care about these things.” (Participant 9)

“In spite of their competency, male nursing officers are often seen as a ‘power lifter’ in the clinical settings.” (Participant 10)

Category 4: Patient Preferences

Some patients express their preference for a female caregiver. This limits male nursing officers’ access to certain patient populations. Female patients reject male nursing officers as "caregivers," as there is awkwardness because of gender and their desire to preserve privacy.

“While proving care to female patients; patients or their family members request for female nursing officers instead of us.” (Participant 13)

“Every time, we need a female assistant during assessment and providing nursing care to the female patients. Married patients’ husbands shun us because of our gender.” (Participant 9)

Category 5: Limited Scope

The administrators limit the skills of male nursing officers by not posting them in the O&G and pediatric departments. The supervisors and ward in-charges occasionally forbid access to these wards during their duty hours. Authorities believed the male nursing officers lacked sufficient knowledge and skills regarding the procedures of these areas because they were not exposed to the O&G ward throughout their learning period.

“For us, the career opportunities are limited, as the O&G department will never accept us.” (Participant 10)

“The hospital authorities didn’t post us to these particular wards and even stated clearly that only female nursing officers can do work in these areas.” (Participant 6)

Theme 3: Initiatives and suggestions to enhance the numbers of male nursing officers

The Odisha government is taking efforts to eliminate discrimination and promote the position of male nursing officers in society. After the COVID-19 pandemic, the government has given them more recognition for their contributions. Recently, the Odisha government has appointed registered male nursing officers as "community health officers (CHO)" in government hospitals following a six-month training program, while female nursing officers have not yet been given this opportunity.

“In the clinical setting, the government also changed the designation of ‘staff nurse’ to ‘nursing officer’, it is a good step; it’s also helping us in our promotion and career development.” (Participant 12)

People should appreciate the contributions of male nursing officers in the society. Senior authorities, parents, and teachers should motivate male students to choose nursing as a profession. The portrayal of unfavorable viewpoints of the nursing profession in films, television shows, and web series must come to an end. Participants expressed that the Odisha government should increase the reservation seat of 10% for admission in the GNM course and that the central government should abolish the gender-based quota in the Nursing Officer Recruitment Common Eligibility Test (NORCET) examination.

“There are only 10% seats reserved for the male students in the GNM course; the government should expand the numbers.” (Participant 8)

“IIn the NORCET examination, 80% seats are reserved for the females and 20% for the boys; due to this, we confront discrimination and challenges.” (Participant 6)

## Discussion

This is the first study in eastern region of India, Odisha, that explores the perceptions and experience of male nursing officers regarding gender inequality in the clinical settings.

This study reveals that male nursing officers encounter challenges such as gender bias, occupational injustice, and social stigma. The results of this investigation corroborated prior studies on men’s experiences as nursing officers. Participants expressed that they had to struggle to get into this profession due to a lack of recognition and comprehension from their own profession as well as the society.

The results of this study demonstrate that due to the physical strength and endurance, male nursing officers were allocated more complex areas such as the ICU, MICU, OT, Cath lab, and emergency. According to a similar study, ICUs and EDs are examples of high-acuity environments where male nursing officers are more often employed [4]. By performing some tasks that require greater physical power, they also support their colleagues. Male nursing officers are ideal for work requiring physical stamina and managing aggressive and agitated patients in psychiatric and emergency departments [14].

Some male nursing officers demonstrate strong technical efficiency and a notable interest in technology, which can enhance patient care processes. Additionally, they have a greater interest in technology and are often posted into the highly technological departments such as endoscopy and OT. A similar study revealed that jobs requiring technical expertise are better suited for male nursing officers [16].

Being a part of RRT and accessible 24x7 in any emergency, male nursing officers are transforming the healthcare system and society. Additionally, they may also show higher endurance levels and proficiency in CPR skills. By being accessible in any emergency situation and 24x7, they are making a contribution to the society and the healthcare system. Accentuating the masculine role in nursing profession, male nursing officers frequently choose high-tech, emergency, critical care or nursing administration positions which require greater physical strength [7].

Male nursing officers serve as role models and an inspiration to both junior nursing officers and students. Participants stated that male nursing officers are more willing to teach juniors the necessary skills and procedures than female nursing officers. It would be advantageous to have more male faculty members and clinical supervisors as role models, as their presence would encourage learning and role socialization [4]. It may be advantageous to have positive male role models in this profession in order to lessen the stigma and promote gender diversity in the workforce [10].

In India and worldwide, there is an increasing demand for male nursing officers. Participants reported that patients of both genders are demanding male nursing officers in the provision of care for their unique traits. Since they are the minority in this profession, the government is also taking initiatives to protect them and encourage male students to pursue careers in the nursing profession. The general shortage in this profession may be addressed by adding more male nursing officers to the workforce, which will certainly enhance the working conditions of the nurses [17].

Occasionally, doctors and other health professionals treat male nursing officers with disdain, assuming that they lack knowledge and skills. However, in some cases, healthcare professionals have shown a lack of respect toward nursing officers, perceiving them as individuals who could not succeed in other medical professions. This reflects a need to address negative stereotypes and promote professional dignity for all nursing roles [8].

Male nursing officers endured prejudice, delayed promotion, and lack of cooperation from higher authorities. They are questioned frequently regarding their job description and contribution in this profession. Participants revealed that they thought about quitting their jobs due to low pay, gender bias, and less job satisfaction, while others revealed that they attempted to get a job in government with the hope for a brighter future. They also pointed out that the nursing profession is seen as a female profession. The general public continues to perceive nursing as a female-dominated profession [8]. Because of long-standing stigma, it is difficult for men to fit into the predominantly female nursing culture, which impairs their ability to provide quality patient care and ultimately decreases their level of job satisfaction [10]. Compared to male careers, nursing is still perceived as a job with no skills, power, and social importance [8].

Strengths

The descriptive phenomenological approach allowed for in-depth exploration of male nursing officers’ perceptions and experiences, capturing nuances that quantitative approaches may overlook. The chosen methodology was well-suited to the research question, enabling participants to share their experiences in their own words. Interviews continued until thematic saturation was reached, enhancing credibility of the findings. Ensured participants had relevant clinical experience (≥2 years) to provide meaningful insights.

Limitations

The small sample size (14 participants) and focus on two tertiary care hospitals in one district (Khordha, Odisha) limit the applicability of findings to broader populations. As the primary data collector, the first author’s background in nursing could have influenced the framing of questions and interpretation of responses; reflexive practices are essential to mitigate this. Interviews conducted in Odia/Hindi and translated into English may risk subtle meaning loss. The transcripts were not returned to the participants for modifications or comments. In order to verify the accuracy of the transcripts, transcription of the interviews verbatim was performed after the acquisition of raw data. The transcript’s authenticity was thoroughly reviewed and cross-checked. It is recommended that further studies involving more districts of Odisha should be conducted using a mixed-method approach.

Methodological consideration

Research and source triangulation was adopted in this investigation to increase the study’s credibility. In order to assure the consistency of the study’s findings, the authors cross-checked the coding, themes, and descriptions. The transcript’s authenticity was thoroughly reviewed and cross-checked. The authors come from diverse educational and professional backgrounds, such as public health and community health nursing, which facilitated in expanding the scope of analysis and interpretation of results. Although this study was conducted in Odisha, the findings might be also useful in other similar settings.

## Conclusions

The themes that arose from this study indicate that, despite being a minority in nursing, male nursing officers possess opportunities and advantages and that the organizations and the government acknowledge their contributions to the healthcare system. The nursing profession is still predominantly comprised of women. Male participation percentages in nursing are exceptionally low, which has a direct connection to prevailing prejudices and beliefs against men who work in this profession where women dominate.

## References

[1] (2025). State of the world's nursing 2020: investing in education, jobs and leadership. https://www.who.int/publications/i/item/9789240003279.

[2] Popper-Giveon A, Keshet Y, Liberman I (2015). Increasing gender and ethnic diversity in the health care workforce: the case of Arab male nurses in Israel. Nurs Outlook.

[3] Achora S (2016). Conflicting image: experience of male nurses in a Uganda's hospital. Int J Afr Nurs Sci.

[4] Woo BF, Goh YS, Zhou W (2022). Understanding the gender gap in advanced practice nursing: a qualitative study. J Nurs Manag.

[5] Appiah S, Appiah EO, Lamptey VN (2021). Experiences and motivations of male nurses in a tertiary hospital in Ghana. SAGE Open Nurs.

[6] Zeb H, Younas A, Rasheed SP, Sundus A (2020). Lived experiences of male nurse educators: an interpretive phenomenological inquiry. J Prof Nurs.

[7] Saleh MY, Al-Amer R, Al Ashram SR, Dawani H, Randall S (2020). Exploring the lived experience of Jordanian male nurses: a phenomenological study. Nurs Outlook.

[8] Cui N, Wang R, Song F, Jin J (2021). Experiences and perceptions of male nursing students in a single-sex class: a qualitative descriptive study. Nurse Educ Pract.

[9] Sharma S, Mudgal S, Rawat R (2022). Patient perception towards males in nursing profession in India: a single center, cross-sectional survey. Int J Afr Nurs Sci.

[10] Kronsberg S, Bouret J, Brett A (2017). Lived experiences of male nurses: dire consequences for the nursing profession. J Nurs Educ Pract.

[11] Smiley R, Ruttinger C, Oliveira C (2021). The 2020 National Nursing Workforce survey. J Nurs Regul.

[12] (2025). CAT notice to AIIMS, govt as plea says 80% quota for women nurses is ‘gender discrimination’. https://theprint.in/india/governance/cat-notice-to-aiims-govt-as-plea-says-80-quota-for-women-nurses-is-gender-discrimination/492144/.

[13] Liu HY, Li YL (2017). Crossing the gender boundaries: the gender experiences of male nursing students in initial nursing clinical practice in Taiwan. Nurse Educ Today.

[14] Yip YC, Yip KH, Tsui WK (2021). Exploring the gender-related perceptions of male nursing students in clinical placement in the Asian context: a qualitative study. Nurs Rep.

[15] Hay K, McDougal L, Percival V (2019). Disrupting gender norms in health systems: making the case for change. Lancet.

[16] Adeyemi-Adelanwa O, Barton-Gooden A, Dawkins P, Lindo JL (2016). Attitudes of patients towards being cared for by male nurses in a Jamaican hospital. Appl Nurs Res.

[17] George C, Bhatti F (2019). The voices of male nurses in Kerala: career choice and satisfaction. Space Cult India.

[18] Ayala RA, Holmqvist MT, Messing HB, Browne RF (2014). Blessed art thou among women: male nursing students and gender inequalities in Chile. Nurse Educ Today.

